# Development of a Moisture-in-Solid-Insulation Sensor for Power Transformers

**DOI:** 10.3390/s150203610

**Published:** 2015-02-04

**Authors:** Belén García, Diego García, Guillermo Robles

**Affiliations:** 1 Department of Electrical Engineering, Universidad Carlos III de Madrid, Avda de la Universidad 30, Leganés, Madrid 28911, Spain; 2 School of Electrical and Electronic Engineering, Edificio 356 - Ciudad Universitaria, Meléndez, Calle 13 No 100-00, Cali 760032, Colombia

**Keywords:** dielectric response, power transformer, moisture, solid insulation, moisture sensor, FDS, moisture monitoring

## Abstract

Moisture is an important variable that must be kept under control to guarantee a safe operation of power transformers. Because of the hydrophilic character of cellulose, water mainly remains in the solid insulation, while just a few parts per million are dissolved in oil. The distribution of moisture between paper and oil is not static, but varies depending on the insulation temperature, and thus, water migration processes take place continuously during transformers operation. In this work, a sensor is presented that allows the determination of the moisture content of the transformer solid insulation in the steady state and during the moisture migration processes. The main objective of the design is that the electrodes of the sensor should not obstruct the movement of water from the solid insulation to the oil, so the proposed prototype uses a metallic-mesh electrode to do the measurements. The measurement setup is based on the characterization of the insulation dielectric response by means of the frequency dielectric spectroscopy (FDS) method. The sensitivity of the proposed sensor has been tested on samples with a moisture content within 1% to 5%, demonstrating the good sensitivity and repeatability of the measurements.

## 1. Introduction

Power transformer reliability is essential for the adequate operation of power systems. Some of the main causes of transformer failures are related to their electrical insulation. These failures usually result in huge economical costs for the electric companies in terms of damage to infrastructure and penalization for interruptions of the electricity supply [1].

Water is one of the most damaging agents for transformer cellulosic insulation. Its presence increases the aging rate of the insulation, reduces its dielectric margin and decreases the partial discharge inception voltage [2]. These phenomena increase the probability of unexpected failures, so being able to estimate the water content of transformer solid insulation is highly desirable to make adequate decisions related to maintenance operations, as well as to predict potential failure conditions [3,4].

Different methodologies have been proposed to determine the moisture content of transformer solid insulation. Some of them are based on the use of equilibrium charts [5–7], which allow the estimation of the water content of the solid insulation if the temperature and the water content of oil are known. However, IEEE Standard C57.106.2006 discourages the use of this kind of direct calculation, as the charts are only valid under equilibrium conditions, and their use might lead to errors in the estimation of moisture of up to 200% [8].

Several authors propose the use of oil-moisture probes combined with mathematical models to characterize the dynamics of the water exchange between paper and oil [9–12]. These models can provide a good estimation of the moisture on the surface of the solid insulation; however, not so precise information of the moisture in the inner part of the insulation can be obtained by using them. It must be considered that the time constants of water-migration between paper and oil are much longer that the thermal cycles inside the transformer, and as a consequence, the water on the surface of the paper mainly participates in the exchange.

Alternatively, the methods based on the determination of the dielectric response of the transformer insulation have been pointed out by Cigre as the most suitable to assess the moisture content of paper and pressboard [13]. Dielectric response methods are founded on the fact that when a dielectric material is subjected to an electric field, its elemental dipoles are oriented in the direction of the field. Dipole orientation is not instantaneous, but has a certain delay. The so-called dielectric response function, which characterizes the polarization transient, depends on different aspects, such as the material nature or the geometry of the object under study, but it also depends on the water content it contains.

Different measuring schemes might be currently used to obtain the dielectric response of the insulation in the time or the frequency domains, and commercial equipment is available to be used in the field. These techniques are accurate and lead to reliable estimations of the average moisture content of the solid insulation. However, they should be applied off-line, since the current measuring schemes involve short circuiting the transformer windings to be used as electrodes.

In this paper, a capacitive sensor is proposed to determine the moisture content of the transformer solid insulation while the transformer is in service. The difference of the proposed scheme with regard to classical approaches is that, in this case, the measurements are done on a test object composed of Kraft paper. The test object is fit inside the transformer tank, where it is subjected to temperature profiles similar to those of transformer solid insulation, and as a consequence, it describes a moisture dynamics similar to that of transformer solid insulation. The dielectric response of the object is measured to determine its moisture content, and this indication is used to estimate the distribution of moisture of the transformer solid insulation.

To define the geometry of the sensor, it is important to consider that the migration of moisture between paper and oil should not be obstructed by the plates of the capacitive probe. To this aim, a metallic mesh is used as the external electrode, while a solid core of steel is used as the inner electrode of the capacitor.

The advantage of the presented measuring scheme is that it allows having a continuous measurement of the water content of the solid insulation. The sensor is not proposed to be an alternative to the off-line dielectric measurements, but to complement the information provided by them between transformer discharges. The new measuring scheme could also be used during moisture dynamics experiments, which are usually very complex and time consuming when the traditional methods based on periodical direct determinations of the moisture content of the insulation are applied [14,15].

In this work, a set of studies are conducted to evaluate the proposed design. The measurements were done using mesh electrodes with different aperture sizes, and the influence of the mesh electrode in the moisture dynamics was evaluated, as well. The sensibility of the sensor was also tested using specimens prepared with different moisture contents, which were evaluated with the proposed measuring scheme. The test objects were characterized using the commercial equipment IDA 200, which measures the frequency dielectric response of the objects (FDS).

## 2. Design of the Sensor

### 2.1. Measuring Principle

As explained before, the objective of the sensor proposed in this work is to monitor the dynamics of moisture in the transformer solid insulation on-line.

The insulation of power transformers is composed of two materials with very different affinity to moisture; while the cellulosic materials that form the solid insulation are very hydrophilic, the mineral oil, which is generally used as the liquid insulation, is highly hydrophobic. As a consequence, most of the water of a transformer is retained by the solid insulation. However, the distribution of moisture between paper and oil is not static, but depends on the transformer operating conditions.

The water saturation level of paper and oil have an opposite tendency when the temperature raises. While the solubility of water in oil increases with the temperature, the capacity of paper to adsorb it diminishes. As a consequence, when the transformer becomes hotter, the oil is able to absorb a larger amount of water from the paper [2] and a migration of moisture from paper to oil appears. The direction of water flow changes if the temperature diminishes.

The temperature inside the transformer varies along the day, because the fluctuations of the power demand are directly related to the winding power losses. These temperature variations cause moisture migration processes between paper and oil during the transformer's normal operation. Additionally, the temperature distribution throughout the transformer windings is not homogeneous, and as a consequence, an inhomogeneous water distribution appears within the transformer insulation. While warmer parts of the insulation on the top of the windings remain dryer, higher amounts of water are concentrated in the colder parts of the transformer.

Different authors [5–7] have developed moisture charts that allow determining the distribution of moisture between paper and oil for a given temperature in steady-state conditions. It should be noted that the time constants of thermal and moisture dynamic processes are very different, and as a consequence, the moisture equilibrium between paper and oil will never be attained during the normal operation of the transformer.

The aim of this work is to evaluate a new measurement scheme that allows the continuous estimation of the moisture content of the transformer solid insulation. The technique is based on the characterization of a probe constituted by a coil of Kraft paper allocated between two metallic plates forming a cylindrical capacitor. The probe is fit in the transformer tank and operates immersed in the transformer oil, where it is subjected to temperature profiles similar to those of the solid insulation. It should be noted that, as the probe is constituted of the same material that composes the solid insulation of the windings, the time constants of water diffusion and the equilibrium conditions of the specimen that is characterized will be similar to those of the insulation of the transformer winding.

The installation of the probe in a transformer would be similar to the installation of the moisture-in-oil probes that are widely used nowadays. As in that case, the probe should ideally be inserted into the hot oil flow and as close as possible to the winding [12]. If the probe is installed in a different position or if the distribution of moisture throughout the winding needs to be estimated, further calculations might be done considering the distribution of temperature on the winding and using a moisture diffusion model.

The moisture content of the Kraft paper coil is derived from the measurement of its dielectric response. The dielectric response measurement is measured in the frequency domain at a voltage below 10 V, which allows one to make the measurement safely while the transformer is in service.

Further work is being carried out to develop the additional models necessary to include the previous aspects in a complete monitoring system where other variables, such as temperature, moisture-in-oil and load, are measured, to allow a precise calculation of the moisture content of the solid insulation throughout the height of the winding.

### 2.2. Geometry of the Sensor

To define the geometry of the sensor, it must be considered that the processes that are being characterized involve moisture movement between solid and liquid insulation, and to avoid the influence of the electrodes on the migration processes, they should not obstruct the contact surface between both materials. For this reason, the sensor was designed with a mesh-electrode that guarantees the movement of water through it and takes all of the previous aspects into account. These processes of adsorption and desorption of moisture from oil to paper, and *vice versa*, have a certain hysteresis that is generally neglected, because it represents small variations [2]. This hysteresis is exclusively related to the physical processes in the paper, but not the measurement device.

The measuring principle of the sensor is based on determining the variation of the capacitance (C′) and the dielectric losses (C″) of a Kraft paper coil with frequency. The best approach to accomplish this used capacitive sensors, where the relationship between the permittivity of the dielectric and the impedance is a constant that depends on the geometry of the sensor. Then, two metallic plates can be connected to a power supply and to the ground, respectively, and the dielectric under test can be placed between them to form a capacitor.

The variation of the impedance can be calculated by measuring the applied voltage and the current at different frequencies. Most measuring schemes are based on this design, but some disadvantages are found when measuring moisture variations in impregnated samples. The most important objection is that the plates are solid and do not allow the flow of moisture if the dielectric under test is heated. Then, at least one of the sides of the capacitor must be a metallic mesh, so moisture can migrate from paper to oil.

The objective is to have a metallic structure so dense, that the capacitance is not negligible and can be easily measured, but with an aperture size wide enough to allow the flowing of moisture. Several meshes with different aperture sizes were cut into rectangles 16 cm long and 6 cm wide and tested to evaluate their performance (Table 1). The material is stainless steel AISI (American Iron and Steel Institute) 304.

The other problem is to ensure the repeatability of the measurements, designing a geometry of the sensor that remains invariant during the whole process and attaching the paper tightly, so that the capacitance does not change. The proposed sensor is based on a cylindrical capacitor instead of a plane capacitor. The dielectric under test is wound around a cylindrical holder and fixed by cable ties. Then, the paper attached to the structure is subjected to the impregnation process explained in Section 3.

Finally, the metallic mesh is wrapped around the paper to complete the capacitor, and the prototype is ready to be tested by applying voltage to the mesh and the metallic bulk. This step is carried out using an insulation diagnostics system, IDA 200, that calculates the dissipation factor, tangent *δ* and the capacitance and dielectric losses of the object for a range of frequencies from 0.1 mHz to 1 kHz.

**Figure 1.**
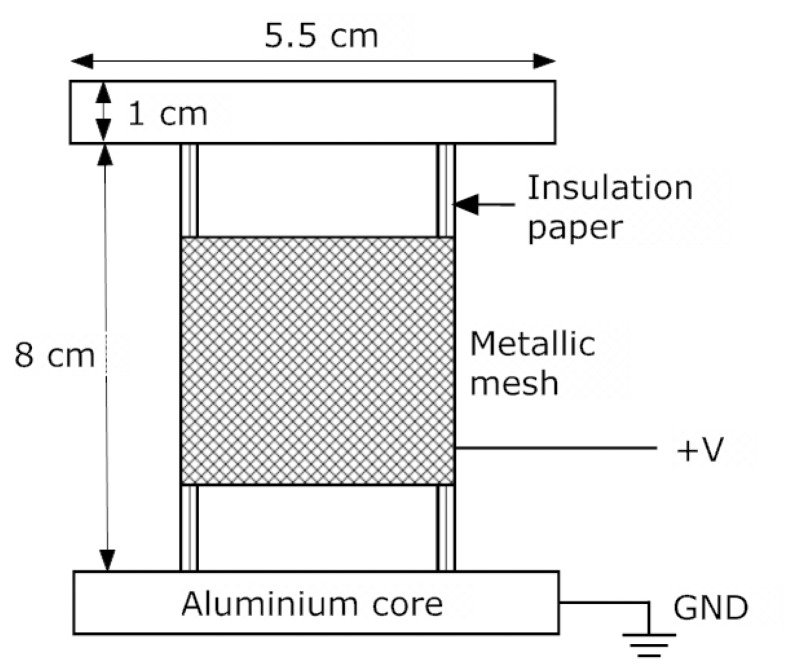
Layout and dimensions of the prototypes with the metallic mesh and the layers of insulation paper.

**Figure 2.**
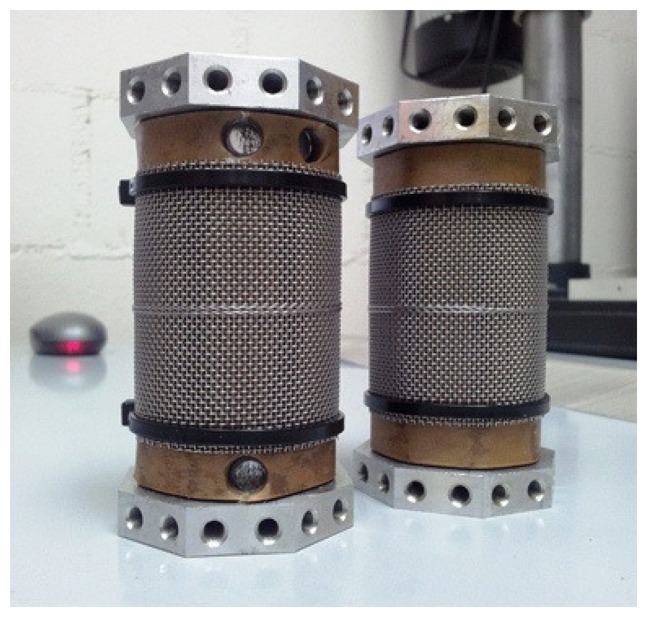
Two prototypes with impregnated paper and metallic mesh belts. The left specimen has three holes where samples have been take to be analyzed with the Karl Fischer method.

Figure 1 shows the scheme of the prototype, including its dimensions, and Figure 2 shows a picture of two of the prototypes. As can be seen, the prototype on the left has some holes on its top and bottom sections that correspond to paper samples that where taken during the experiments to determine the exact moisture content by direct methods, using the Karl Fischer method.

The Kraft paper used to manufacture the prototypes is 0.1 mm thick and Type p.4.1, according to the standard IEC 60641-1 [16].

## 3. Experimental Evaluation of the Design

To evaluate the adopted geometry, the following aspects should be checked:

1. The mesh electrode should allow the performance of coherent and repetitive FDS measurements.
2. The presence of the mesh-electrode should not affect the moisture distribution of the test specimen.
3. The FDS measurements obtained with the proposed sensor should be sensitive to the changes of moisture in the sample.

### 3.1. Effect of Using a Mesh-Electrode and Selection of the Aperture Size

As explained before, tests were done using meshes with different aperture sizes (Table 1) to evaluate which of them were suitable to obtain reliable measurements.

**Figure 3.**
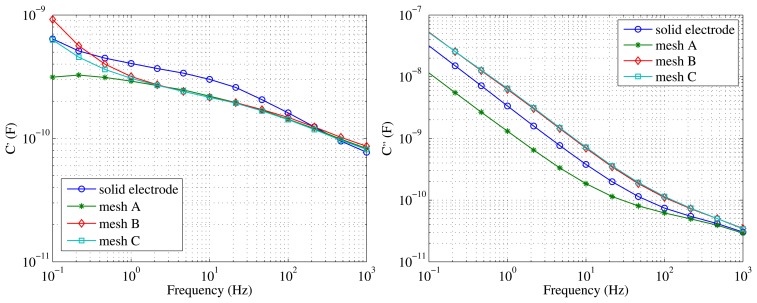
Capacitance (C′) and dielectric loss (C″) measured with different electrodes.

To this aim, an initial FDS measurement was performed on a test specimen by fitting a solid aluminum electrode around it. Then, the external electrode was removed, and the measurements were repeated over the same test specimen, but using the different meshes defined in Table 1. The results of those measurements are displayed in Figure 3.

The results show that the three tested meshes could be used in the sensor, because they detect changes in the dielectric parameters with frequency, so there is indeed a measurable capacitance between electrodes. Two consecutive measurements were performed with each mesh on the same object, finding almost identical curves in the three cases.

The measurements taken with Mesh A are clearly different when compared with the rest of the configurations. In the case of the measurement of C″, lower values of dielectric losses are obtained. Additionally, the measurements of C′ at low frequencies with Mesh A take very long times, and they seem to be less reliable than those taken with the rest of the electrodes. As for Meshes B and C, they give the same results when measuring C″ and are very similar in the case of C′, the differences being at low frequencies. Finally, Mesh B was considered the best candidate, because it had a larger aperture than Mesh C, and it would interfere less with water migration from the paper to oil, keeping the same conditions in the covered area as in the area that is directly in contact with the oil.

### 3.2. Effect of the Mesh-Electrode in the Moisture Distribution of the Sample

Test specimens were prepared with a high moisture content by exposing them to an environment of controlled relative humidity (RH). Before the humidification of the specimens, the mesh-electrode was fitted to the paper, covering part of its central surface (Figure 2), while the upper and lower sections of the paper remained uncovered.

After the humidification process, some paper samples were taken at different heights of the test specimen and analyzed with the Karl Fischer method to determine their moisture content [17].

The moisture distribution in height can be seen in Table 2. Concretely, four different samples were taken at different heights in a test specimen prepared with a moisture content of about 3%. Two of these samples were in the area covered by the mesh and the other two in the uncovered area. Considering that the Karl Fischer method has a uncertainty of about 0.3% in the determination of the moisture content of paper [17], the differences found between the different samples can be considered negligible.

Measurements were also made taking samples at three different depths of the specimen inside and outside the mesh: a sample of external insulation (*i.e.*, in direct contact with air), another at an average depth and one of the paper in contact with the aluminum core. As can be seen in Table 3, the moisture distribution can be also considered homogeneous, and no influence of the mesh can be discerned.

From the previous analysis, it can be concluded that the presence of the chosen mesh does not affect the movement of water in the insulation. This conclusion seems logical given that the size of the water molecule is in the range of Å, while the size of the mesh is 0.75 mm.

### 3.3. Sensitivity of the Sensor

The sensitivity of the sensor to the variation of the moisture of the paper was also determined. To this aim, measurements were performed on test specimens with different moisture contents. Four specimens were prepared with moisture contents of 1.8%, 3.6%, 4% and 5.3% with the structure shown in Figure 2 using a drying oven and different saline solutions, according to the standard ASTM E104-02 [18]. The moisture concentration of the specimens was determined with Karl Fischer titration after the experiments.

**Figure 4.**
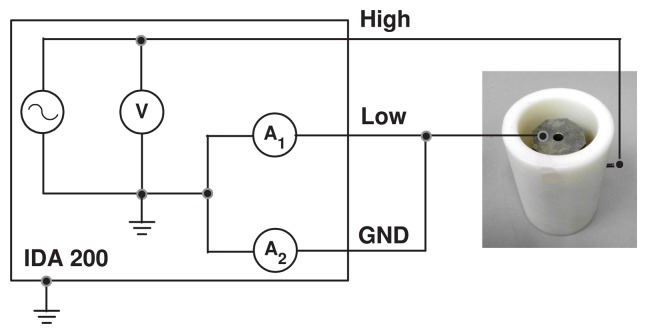
Measuring scheme.

The samples were introduced into a Teflon container filled with new and dry mineral oil, as would be done in a real transformer. FDS measurements were carried out using the scheme shown in Figure 4, the applied voltage being 10 V for all cases. The process lasts in the range of tens of minutes, which is negligible compared to the time constant of the humidity drift between paper and oil. This ensures that the moisture in the samples is constant during the measuring process. The oil used in this work was Nytro Taurus, manufactured by Nynas.

**Figure 5.**
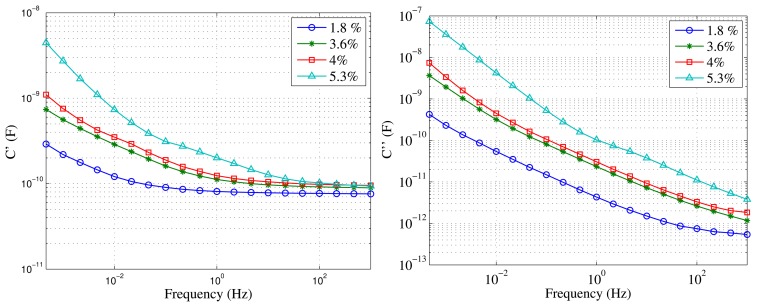
C′ and C″ measured with Mesh B over samples with different moisture levels.

Figure 5 shows the comparison of the FDS measurements on the different specimens. Their dielectric responses are very different, especially at lower frequencies, as expected. It can be seen how small differences of moisture can also be distinguished by the sensor. The measurements where done in the frequency range of 0.1 mHz to 1 kHz, although shorter measurements would be also valid to make the diagnosis.

**Figure 6.**
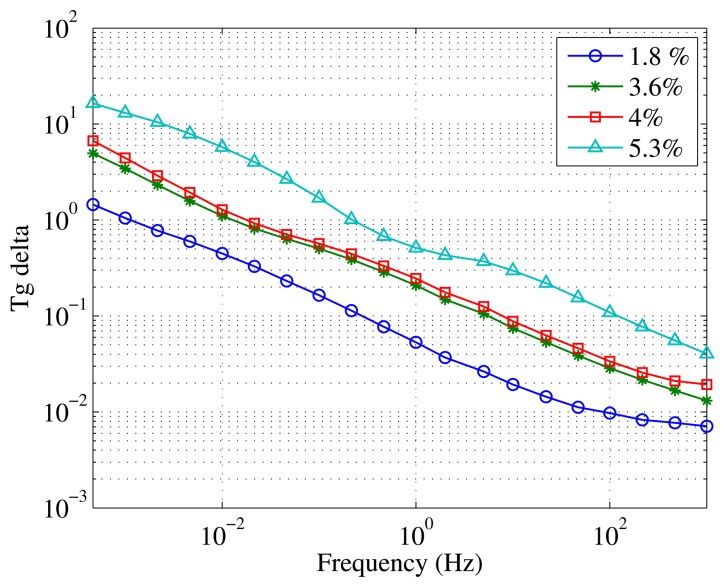
Tangent *δ* measured with Mesh B over samples with different moisture levels immersed in mineral oil.

Additionally to C′ and C″, tangent *δ* can be considered as a control variable to monitor the moisture content [19]. The commercial FDS measuring equipment available nowadays allows plotting the results of dielectric measurements in C′-C″ graphics or in tangent *δ vs.* frequency. Figure 6 shows the behavior of tangent *δ* of the specimens with different humidities as a function of frequency. As can be seen, tangent *δ* is also a good parameter to use to determine the moisture content of paper.

The values of capacitance, dielectric losses and tangent delta *versus* frequency and humidity are consistent with what is expected from the bibliography [13,20].

The aim of this type of measurement is not obtaining an exact value of the humidity in the paper, but the evolution of the humidity degree, which suggests a maintenance intervention. Nevertheless, the overall inaccuracy of the instrumentation is estimated to be below 1%, as according to the datasheets of the manufacturers, the inaccuracy of the IDA 200 is 0.5% and the precision of the Karl Fisher method is 0.3 % for samples with water content above 1000 *μ*g.

## 4. Variables of Influence and Corrections

### 4.1. Dependency with Temperature

Dielectric measurements are very sensitive to temperature variations. To get reliable estimations of the moisture content, it is basic to consider the temperature of the samples during the measurements. To this aim, the sensor includes a temperature probe that is placed in a hole on the top of the aluminum core.

The temperature dependence of the dielectric measurements has been widely studied [2,13], observing that the changes of temperature cause a shifting of the dielectric response frequency spectra. However, in most dielectric materials, such as oil-paper insulation, temperature does not affect the shape of the dielectric response significantly, which allows one to normalize the measurement data for different temperatures into one single curve, called the master curve.

The shifting of the dielectric response curves with temperature can be expressed with an Arrhenius factor and may be calculated using the equation proposed by Ekanayake in [20]:

(1) $$\text{shift}=\log\left(\omega_{1}\right)-\log\left(\omega_{2}\right)=-\frac{E_{a}}{k\times 2.30258}\left(\frac{1}{T_{2}}-\frac{1}{T_{1}}\right)$$

where *k* is Boltzmann's constant, *E_a_* is the activation energy of the material (typically 1 eV in oil-paper insulation [2]) and *T*_1_ and *T*_2_ are the considered temperatures expressed in Kelvin.

**Figure 7.**
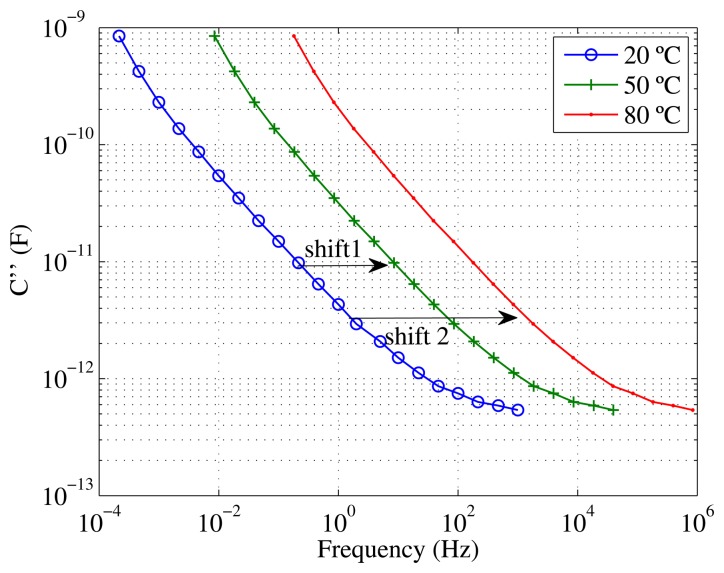
Dielectric losses of the sample with moisture 1.8% measured at 20 °C and calculated for temperatures 50 °C and 80 °C (Shift 1 = −1.5976, Shift 2 = −2.9236).

All of the measured curves provided in this work were obtained at 20 °C. Equation (1) was applied to calculate how the dielectric losses measured on the sample of 1.8% moisture would have changed if the measurements had been taken at temperatures of 50 °C and 80 °C (Figure 7). The same expression might be used to calculate the shifting of other variables, such as capacitance and tangent *δ* with temperature.

### 4.2. Influence of the Relative Humidity of the Environment

The relative humidity (RH) of the environment does not have a significant influence in the measurements of the sensor. The sensor is proposed to be used immersed in oil, which is a very hydrophobic material. Even in humid environments, the contamination of the paper by water taken from oil would be very little. Additionally, the time constants involved in the adsorption of moisture into the paper at room temperature are in the order of months [2], while the measurements with the sensor take about one hour.

The relative humidity of the air during the measurements shown in this work was monitored and had a value within 40%–45%. Moreover, when not used, all samples were kept inside containers with saline solutions that maintain the humidity constant for long periods of time after humidification. The containers, in turn, were stored in a humidity-controlled environment area in the laboratory.

### 4.3. Influence of the Condition of the Oil

When FDS measurements are done in a real transformer, the insulation between the high voltage and low voltage windings, usually called the CHL, measurement, or the insulation between one winding and the ground, usually called CHor CLmeasurements, might be characterized. In all cases, a certain volume of oil is located between the plates of the capacitor that is being measured. The interpretation of the FDS measurements used to be based on the representation of the insulation as an XY model, which should include information related to the condition of the oil and the proportions of oil and paper present between the plates of the capacitor [13].

An advantage of the proposed measuring scheme is that the plates of the capacitor are in direct contact with the solid insulation to be characterized, and thus, the influence of the condition of the oil in the measurements is minimized. This fact significantly eases the interpretation of the measurements. Additionally, as the geometry of the specimen is known with precision, the uncertainty associated with the estimation of the proportions of the different materials is minimized

**Figure 8.**
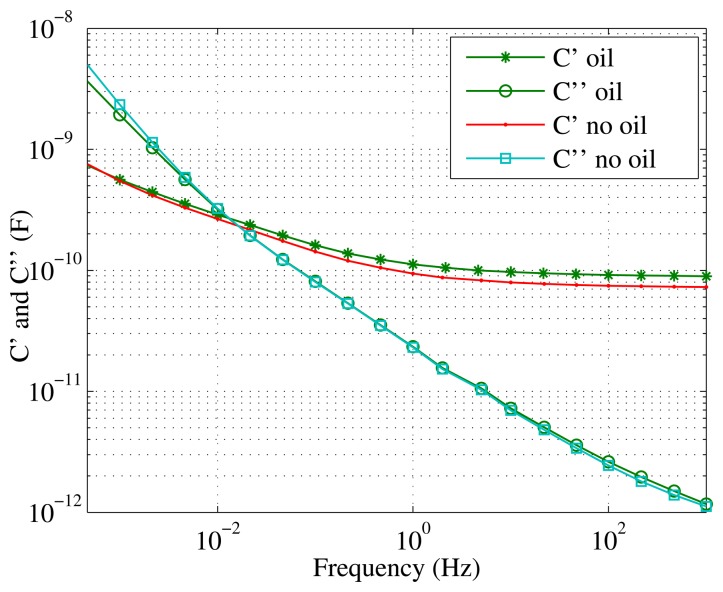
Capacitance and dielectric losses measured with the probe immersed in oil and surrounded by air. Measurements were done for a sample with a moisture content 3%.

Figure 8 compares the measurements obtained on a test specimen when it is immersed in the tank of oil shown in Figure 4 *vs.* the measurements when the object is measured out of the tank and surrounded by air, although the paper is impregnated with oil. As can be seen, very little difference appears between both cases.

### 4.4. Influence of the Condition of the Paper

Although several experiments have demonstrated that the dielectric response of a transformer is influenced by the aging degree of its insulation [21–23], different authors agree on the fact that it is not easy to distinguish between the effect of the condition of the paper from the effect of an increase of its moisture content. Currently, most of the applied interpretation schemes do not consider the effect of the condition of the paper, adding uncertainty to the estimations of the moisture content [24,25].

In the proposed scheme, dielectric measurements are taken on a specimen composed of new paper, and so, the interpretation of the measurements of the sensor will be more easy. The further estimation of the moisture content throughout the transformer insulation is done by means of theoretical models, where the variations of the diffusion coefficient with the aging condition of the paper and the differences in water affinity between new and aged paper are not so difficult to incorporate.

Further investigations are being carried out on this point.

## 5. Conclusions

Methods based on the characterization of the dielectric response have been used for years to estimate the moisture content in transformer solid insulation in field conditions. Nowadays, different commercial equipment is available to make such measurements based on the time domain or the frequency domain analyses. At present, all of the dielectric response measuring schemes require the transformer to be out of service.

In this paper, a sensor is proposed to determine the moisture content of solid insulation in real transformers while the equipment is in service. The sensor is based on the characterization of the dielectric response of a Kraft paper coil that would be fit inside the transformer tank and subjected to the same conditions as the transformer solid insulation.

One of the electrodes has been substituted by a metallic mesh, so that it does not affect the moisture migration processes between paper and oil that continuously take place during transformer operation.

The proposed scheme has been validated with an in-depth study that evaluates the design and the measuring principle of the sensor. Firstly, the selection of the mesh electrode has been justified and tested. Measurements were carried out using meshes with different aperture sizes and also using a solid electrode. Then, the effect of the selected mesh in the moisture distribution of the coil was evaluated using the Karl Fischer method. To this aim, a test specimen was subjected to a humidification process with the mesh on, and the resulting moisture content of the areas covered with the mesh and the uncovered ones were compared in longitudinal and radial directions.

The sensitivity of the sensor was proven on test specimens prepared with different moisture contents and using the FDS commercial equipment, IDA 200, to determine the dielectric response in the frequency domain, with the samples immersed in mineral oil. The sensor demonstrated good sensitivity when these tests were done, allowing us to assume that it will be able to distinguish moisture contents within 1% an 5% with enough accuracy. The values of capacitance *versus* frequency and humidity were consistent with the values reported in the bibliography.

The proposed sensor could be a good complement to the off-line dielectric response measurements, as it would allow one to have information between consecutive discharges. Moreover, the geometry and properties of the materials that compose the sensor are perfectly characterized, which eases the interpretation of the measurements.

The sensor proposed in this work is a prototype. In the future, its dimensions and constructive materials will be revised to ease its integration into a real on-service transformer. Additional work is also being done to adjust the dynamic models that will complement the information given by the sensor, allowing one to calculate the moisture distribution throughout the transformer winding.

## Figures and Tables

**Table 1.** Types of metallic meshes tested.

**Mesh Denomination**	**Aperture Size (mm)**
Mesh A	1×1
Mesh B	0.75 × 0.75
Mesh C	0.45 × 0.45

**Table 2.** Distribution of moisture in a test specimen along its height.

**Sample Ubication**	**Moisture Content (%)**	
	**Outside the Mesh**	**Inside the Mesh**
Top sample	3.4	3.1
Bottom sample	3.5	3.3

**Table 3.** Moisture content at different depths of the test specimen.

**Sample Deepness**	**Moisture Content (%)**	
	**Outside the Mesh**	**Inside the Mesh**
Inner sample	3.1	3.0
Central sample	2.9	3.0
Outer sample	2.9	3.0
